# Durable clinical remission of a skull metastasis under intralesional Viscum album extract therapy: Case report

**DOI:** 10.1002/hed.25320

**Published:** 2018-06-09

**Authors:** Paul Georg Werthmann, Roman Huber, Gunver Sophia Kienle

**Affiliations:** ^1^ Institute for Applied Epistemology and Medical Methodology (IFAEMM) at the University of Witten/Herdecke Freiburg Germany; ^2^ Center for Complementary Medicine, Institute for Infection Prevention and Hospital Epidemiology, Medical Center – University of Freiburg, Faculty of Medicine University of Freiburg Freiburg Germany

**Keywords:** mistletoe, rectal cancer, skull metastasis, thyroid carcinoma, Viscum album

## Abstract

**Background:**

Skull metastases are rare, they can eventually cause pain, and can invade the brain. Viscum album extracts (VAEs) are used as an adjuvant treatment in cancer.

**Methods and Results:**

A 68‐year‐old patient with rectal cancer presented with lung metastases, and metastases to multiple bone sites, the chest wall, and the liver were later identified. Histological examination of one of the bone lesions revealed an additional thyroid carcinoma. An osteolytic parietal bone lesion progressed to a painful metastasis of the skull despite radiotherapy and chemotherapy. The VAEs were applied weekly into the metastasis, followed by pain relief and softening of the lesion. The lesion partially regressed (>50%) after 8 months of continued VAE treatment and remained stable for 2 years.

**Conclusion:**

This case shows a durable clinical remission of a skull metastasis under VAE. Further investigations of intratumoral VAE treatment seem worthwhile—especially in symptomatic skull metastases not responding to radiotherapy or systemic therapies.

## 1 INTRODUCTION

Metastasis to the skull is a rare event, occurring in various cancers. Although metastasis of the skull base carries the risk of cranial nerve dysfunction, metastases of the calvarian skull are mostly asymptomatic but can cause pain. Either location carries the risk of invasion into the brain. Radiotherapy can be effective, and surgical excision is sometimes used with mixed results^1^; however, clinical studies for comparative treatment options or evidence‐based practice guidelines are not available.^2^

Viscum album L. grows as a hemiparasitic shrub (European mistletoe) on different host trees and contains a variety of biologically active compounds, particularly mistletoe lectins and viscotoxins.^3^ Viscum album extracts (VAEs) and their compounds show antineoplastic activities, including cytotoxic and apoptogenic effects, immune stimulation, downregulation of tumor genes, inhibition of tumor cell migration, and neo‐angiogenesis.^3^, ^4^, ^5^, ^6^, ^7^ Various standardized commercial VAEs are available in injectable preparations (usually used subcutaneously but occasionally intravenously or intratumorally).^8^ Clinical trials found an improved quality of life in patients with cancer treated with VAE and longer survival in patients with advanced pancreatic cancer.^9^, ^10^, ^11^ Remission of various tumors has been described in case reports and small trials.^12^, ^13^, ^14^, ^15^, ^16^, ^17^, ^18^, ^19^, ^20^ Frequent side effects of VAE treatment includes self‐limited, dose‐dependent, inflammatory local skin reactions and flu‐like symptoms. Occasionally, pseudo‐allergic reactions can occur. Otherwise, VAE therapy is safe, even at higher doses.^21^

## 2 CASE REPORT

A 68‐year‐old woman was diagnosed with deep rectal cancer with lung metastases. She was of normal body weight, was a nonsmoker, had a normal diet with low meat consumption, and was physically active (swimming, hiking, and tennis). With regard to family medical history, her sister had gastric cancer, and her father had diabetes.

The primary tumor was excised via deep anterior rectum resection and classified as moderately differentiated adenocarcinoma with expression of carcinoembryonic antigen and specifically p53 (pT2, pN1 [2/9], cM1, and G2). After surgery, a chemotherapy regimen with 4 cycles of folinic acid, 5‐fluorouracil, irinotecan hydrochloride (FOLFIRI) was started and led to stable disease; the regimen was then changed to 2 cycles of folinic acid, fluorouracil, and oxaliplatin (FOLFOX), again resulting in stable metastatic lesions. Epidermal growth factor receptor testing was positive; therefore, the patient was treated with cetuximab and irinotecan, which had to be stopped after the first cycle due to dermatologic side effects; the number and size of pulmonic metastases remained unchanged under this treatment.

A year after the initial diagnosis, osteolytic bone metastases were found in the parietal bone and left pubis. The pelvic metastasis was treated with radiation (36 Gy). A palliative chemotherapy regimen with bevacizumab, irinotecan, fluorouracil, and leucovorin had to be stopped after 2 cycles because of elevated liver enzymes and a recurrence of hepatitis C; additionally, diabetes mellitus was found. The cancer lesions remained stable for 10 months, when new metastases of the cervical spine with infiltration of the neuroforamina were found. Radiation of the cervical spine was performed (40 Gy), bisphosphonate therapy was started (zoledronic acid 4 mg every 3 weeks), and another 6 cycles of FOLFOX4 chemotherapy were given. Under these therapies, the existing metastases remained stable, although a new metastasis appeared on the right chest wall. A maintenance treatment with capecitabine was started (1000 mg/m^2^ d1‐d14 2×/d). Two months later, the patient had a pathologic fracture of the right hip and received a hip prosthesis and radiation of the right hip (36 Gy). Surprisingly, pathologic investigation of the hip bone from surgical replacement showed a metastasis of a follicular thyroid carcinoma; as the thyroid of the patient had been nearly completely removed in a stromal thyroidectomy several years before and the patient's condition was described as palliative, no further investigation regarding this diagnosis was carried out.

At the same time, radiotherapy of the skull with 42 Gy was performed due to progression of the osteolytic lesion of the parietal bone into a calvarian skull metastasis crossing the suture to the occipital bone. Despite radiotherapy, the calvarian metastasis progressed, as did further metastasis of the head region (frontal left sinus with infiltration of the orbita and occipital). Another round of radiotherapy of the other head metastases was performed (occipital = 40 Gy; frontal sinus = 36 Gy) without response.

**Figure 1:**
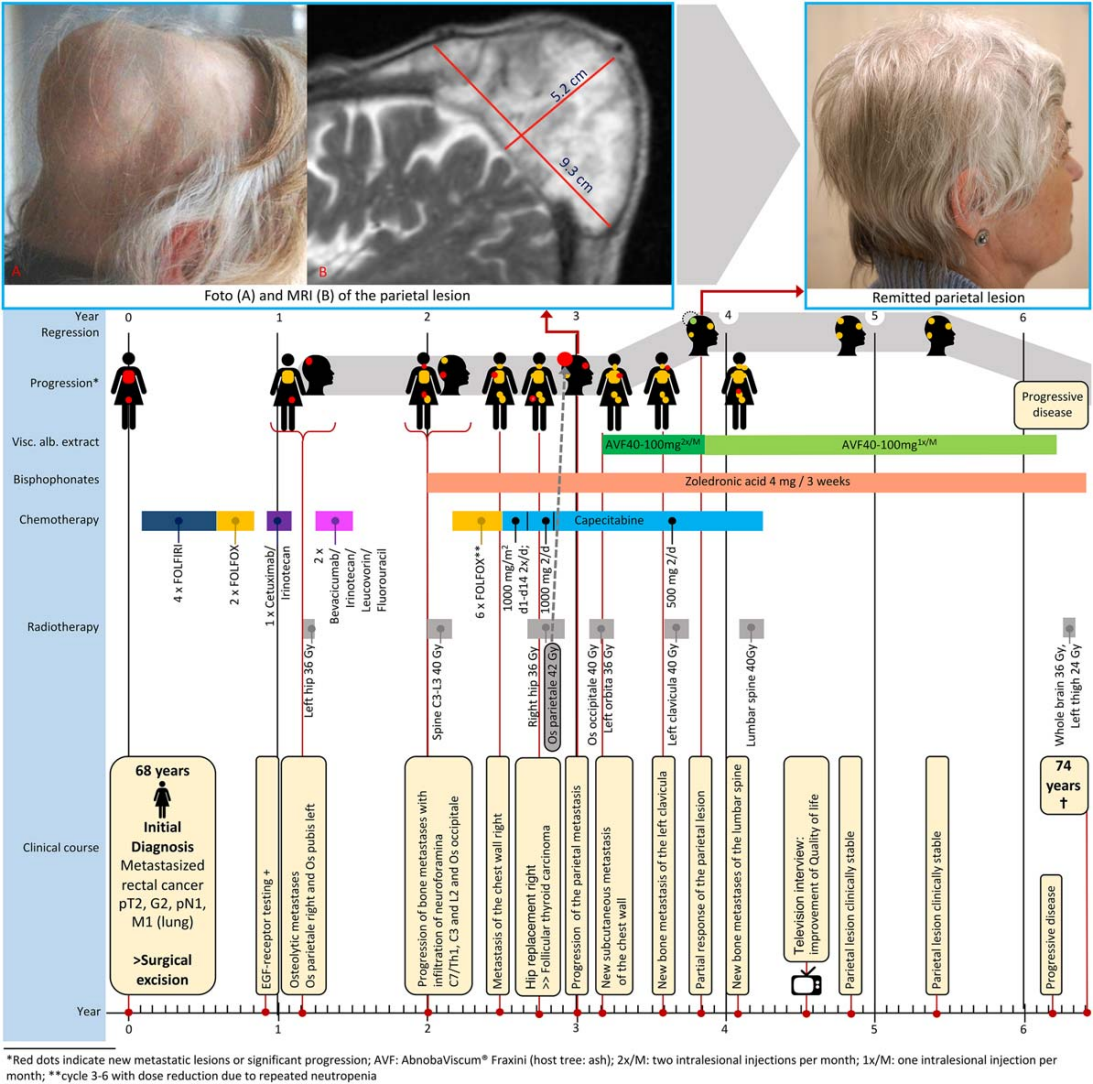
Timeline of the patient with a skull metastasis

At this point, the patient presented with the painful progressing calvarian skull metastasis of 9.3 × 9.3 × 5.2 cm (Figure 1) despite radiotherapy (42 Gy, 4 months before), chemotherapy (capecitabine), and bisphosphonate therapy at the Center for Complementary Medicine at the University of Freiburg, Germany.

The metastasis had necrotic cavities within the tumor mass, from which liquid containing no viable tumor cells could be drawn. Weekly injections of VAE into the calvarian skull metastasis were started (Abnobaviscum fraxini 40‐100 mg). After 2 treatments, the patient reported a reduction of pain and tenderness of the previously tense area of metastasis. As a result of this positive effect, the treatment was continued. The patient experienced mild fever, which was self‐limiting, and eosinophilia up to 47% (normal range <5%), which decreased over time. The size of the metastasis decreased considerably to 6 × 5 × 2 cm (>50%) during the course of treatment and remained stable for 2 years with continued VAE treatment. (Later in the course, the application was reduced to once every 2 weeks.) During the VAE therapy, 1 new bone metastasis appeared at the left clavicle and was treated with radiotherapy. No further metastases appeared, and the other sites of metastases (lung, pubis, and cervical spine) remained stable. After this time, the lesions progressed, and the patient died at the age of 74 years (77 months after initial diagnosis and 39 months after the initiation of intratumoral VAE treatment; for details, see Figure 1).

### 2.1 Preceding and concomitant therapies

The patient was treated with bisoprolol for hypertension, with insulin for diabetes mellitus type 2. She had a medical history of appendectomy and hysterectomy; and hepatic cysts were present.

### 2.2 Patient perspective (from a television interview)

“[My] whole well‐being has changed, and I've got more power to do my things. Beforehand, there were days where I felt completely powerless and I wanted to give up. The mistletoe treatment helped me there.”^22^

## 3 DISCUSSION

We report a case of a woman with a growing calvarian skull metastasis despite chemotherapy (capecitabine) and radiotherapy (42 Gy until 4 months before VAE therapy) who experienced durable partial remission of this metastasis under intralesional VAE therapy. Furthermore, the patient described improvement of her well‐being and psychological state. Other cancer‐specific treatments the patient received included radiotherapy, bisphosphonates, and oral capecitabine during the VAE therapy period. However, as these treatments were already present during progression of the skull metastasis and the patient experienced immediate pain relief after the first VAE treatments, we presumed that the VAE treatment contributed to the partial remission of the calvarian skull metastasis.

Skull metastases are rarely diagnosed clinically and often appear late in the course of cancer progression or are only found at autopsy.^2^ Therapeutic options are often limited by the patient's diminished health or are judged as secondary if the skull metastasis does not lead to functional limitations. Treatment options include systemic anticancer agents, radiotherapy, and—rarely—surgical removal. Pain reduction can be achieved via radiotherapy or stereotactic radiosurgery in a large percentage of symptomatic skull metastases (65%‐90%).^2^ The rate of response to systemic agents or surgery is not known; however, the outcome in these cases usually depends on the overall condition of the patient and the stage of the underlying disease.^2^

We believe that this is the first report about a local treatment of a calvarian skull metastasis with VAE with clinical improvement of the patient and partial remission of the metastasis. Case reports about intratumoral applications of VAE showed durable remission in different types of cancer (cutaneous squamous cell carcinoma,^16^ adenoid cystic carcinoma,^17^ breast cancer, Merkel‐cell carcinoma,^14^ cutaneous B‐cell lymphoma,^15^ and malignant melanoma^12^). In these cases, the high cytotoxic, apoptotic, and immunologic properties of VAE may have been pivotal in inducing the remissions. Given the positive results of the course of treatment in our patient, further investigations of VAE as an intratumoral application in skull metastasis seem to be highly worthwhile.

## ACKNOWLEDGMENTS

The authors thank Dr Helmut Kiene for revision of the manuscript. This case report was prepared following the CARE Guidelines.^23^

## INFORMED CONSENT

Informed consent was received from the patient's next of kin for the publication of the report and accompanying images. The patient's next of kin read the submitted version of the report and confirmed its content to the best of his knowledge.

## AUTHORS CONTRIBUTIONS

*Contributed to the case report design:* P.G.W., R.H., G.S.K.

*Physician in charge and provided the patient's information:* R.H.

*Collected and provided the data:* P.G.W., R.H.

*Principle author of the article, had full access to all data, and is the guarantor:* P.G.W.

*Supervised the report and the publication process:* G.S.K.

## CONFLICT OF INTEREST

The Center for Complementary Medicine at the University of Freiburg has received restricted research grants, honorariums, and travel expenses from Iscador AG, Abnoba GmbH, and Helixor GmbH; none of them had any influence on the design, conduction, analysis, and publication of the study. P.G.W. declares no conflict of interest.

## References

[1] Singh M , Ricci JA , Talbot SG , Chiocca EA , Dunn IF , Caterson EJ . Reconstruction of rare skull metastases using free latissimus dorsi flap and the role of preoperative embolization in hypervascular skull tumors. J Craniofac Surg. 2015;26:2289‐2292. 26501975 10.1097/SCS.0000000000002218

[2] Mitsuya K , Nakasu Y . Metastatic skull tumours: diagnosis and management. Eur Assoc NeuroOncol Magazine. 2014;4:71‐74.

[3] Kienle GS , Kiene H . Die Mistel in der Onkologie ‐ Fakten und konzeptionelle Grundlagen. Stuttgart and New York, Schattauer Verlag; 2003.

[4] Büssing A . Mistletoe: the genus Viscum. Amsterdam, The Netherlands, Harwood Academic, 2000.

[5] Podlech O , Harter PN , Mittelbronn M , Pöschel S , Naumann U . Fermented mistletoe extract as a multimodal antitumoral agent in gliomas. Evid Based Complement Alternat Med. 2012;2012:501796. 23133496 10.1155/2012/501796 PMC3485514

[6] Elluru SR , Duong Van Huyen JP , Delignat S , et al. Antiangiogenic properties of Viscum album extracts are associated with endothelial cytotoxicity. Anticancer Res. 2009;29:2945‐2950. 19661299

[7] Singh BN , Saha C , Galun D , Upreti DK , Bayry J , Kaveri SV . European Viscum album: a potent phytotherapeutic agent with multifarious phytochemicals, pharmacological properties and clinical evidence. RSC Adv. 2016;6:23837‐23857.

[8] Kienle GS , Kiene H . Complementary cancer therapy: a systematic review of prospective clinical trials on anthroposophic mistletoe extracts. Eur J Med Res. 2007;12:103‐119. 17507307

[9] Kienle GS , Kiene H . Influence of Viscum album L (European mistletoe) extracts on quality of life in cancer patients: a systematic review of controlled clinical studies. Integr Cancer Ther. 2010;9:142‐157. 20483874 10.1177/1534735410369673

[10] Tröger W , Galun D , Reif M , Schumann A , Stanković N , Milićević M . Viscum album [L.] extract therapy in patients with locally advanced or metastatic pancreatic cancer: a randomised clinical trial on overall survival. Eur J Cancer. 2013;49:3788‐3797. 23890767 10.1016/j.ejca.2013.06.043

[11] Axtner J , Steele M , Kröz M , Spahn G , Matthes H , Schad F . Health services research of integrative oncology in palliative care of patients with advanced pancreatic cancer. BMC Cancer. 2016;16:579. 27485618 10.1186/s12885-016-2594-5 PMC4971628

[12] von Laue H‐B . Mistletoe treatment for melanoma brain metastases: a single case In: Skin Cancer and UV Radiation. Altmeyer P, Hoffmann K, Stücker M, eds. Berlin, Heidelberg, Springer Berlin Heidelberg, 1997:1315‐1322.

[13] Kirsch A . Successful treatment of metastatic malignant melanoma with Viscum album extract (Iscador M). J Altern Complement Med. 2007;13:443‐445. 17532738 10.1089/acm.2007.6175

[14] Orange M , Fonseca M , Lace A , von Laue B , Geider S . Durable tumour responses following primary high dose induction with mistletoe extracts: two case reports. Eur J Integr Med. 2010;2:63‐69.

[15] Orange M , Lace A , Fonseca M , von Laue BH , Geider S , Kienle GS . Durable regression of primary cutaneous B‐cell lymphoma following fever‐inducing mistletoe treatment: two case reports. Glob Adv Health Med. 2012;1:18‐25. 10.7453/gahmj.2012.1.1.006 PMC3833476 24278797

[16] Werthmann PG , Sträter G , Friesland H , Kienle GS . Durable response of cutaneous squamous cell carcinoma following high‐dose peri‐lesional injections of Viscum album extracts ‐‐ a case report. Phytomedicine. 2013;20:324‐327. 23394841 10.1016/j.phymed.2012.11.001

[17] Werthmann PG , Helling D , Heusser P , Kienle GS . Tumour response following high‐dose intratumoural application of Viscum album on a patient with adenoid cystic carcinoma. BMJ Case Rep. 2014;2014:pii:bcr2013203180. 10.1136/bcr-2013-203180 PMC4120041 25082867

[18] Orange M , Reuter U , Hobohm U . Coley's lessons remembered: augmenting mistletoe therapy. Integr Cancer Ther. 2016;15:502‐511. 27207233 10.1177/1534735416649916 PMC5739169

[19] Mabed M , El‐Helw L , Shamaa S . Phase II study of viscum fraxini‐2 in patients with advanced hepatocellular carcinoma. Br J Cancer. 2004;90:65‐69. 14710208 10.1038/sj.bjc.6601463 PMC2395314

[20] Mahfouz M , Ghaleb H , Hamza M , et al. Multicenter open labeled clinical study in advanced breast cancer patients. A preliminary report. J Egypt Natl Cancer Inst. 1999;11:221‐227.

[21] Kienle GS , Grugel R , Kiene H . Safety of higher dosages of Viscum album L. in animals and humans ‐ systematic review of immune changes and safety parameters. BMC Complement Altern Med. 2011;11:72. 21871125 10.1186/1472-6882-11-72 PMC3180269

[22] SWR Fernsehen in Baden‐Württemberg . Praxis Dr. Weiß ‐ Heilkräuter. 2008. https://www.swrfernsehen.de/. Accessed January 15, 2018.

[23] Riley DS , Barber MS , Kienle GS , et al. CARE guidelines for case reports: explanation and elaboration document. J Clin Epidemiol. 2017;89:218‐235. 28529185 10.1016/j.jclinepi.2017.04.026

